# Supplementary material for: Global hypo-methylation in a proportion of glioblastoma enriched for an astrocytic signature is associated with increased invasion and altered immune landscape
Source: eLife. 2022 Nov 22;11:e77335. doi: 10.7554/eLife.77335 (PMC9681209; doi:10.7554/eLife.77335)
Supplement: Figure 2—source data 1. [file elife-77335-fig2-data1.zip › Figure_2_source_data_1/Figure_2B/homerResults/motif58.similar.html]

motif58

## Information for motif58

G
C
A
T
C
G
A
T
G
C
A
T
G
C
T
A
C
G
T
A
G
C
T
A
G
C
A
T
C
G
T
A
G
C
A
T
G
C
A
T
G
C
A
T
G
C
T
A
G
C
A
T
G
A
C
T
A
G
C
T
C
G
A
T
C
G
A
T
C
G
A
T
C
A
G
T
C
G
A
T
G
C
A
T
G
C
A
T
G
C
A
T
G
A
C
T
C
G
A
T
G
A
C
T
G
A
C
T
G
C
A
T
G
C
T
A
G
C
T
A
C
G
T
A
C
G
T
A
C
G
T
A
C
G
A
T
C
G
A
T
C
G
A
T
G
C
A
T
G
A
C
T
G
A
T
C
G
A
C
T
  
Reverse Opposite:  

C
T
G
A
C
T
A
G
C
T
G
A
C
G
T
A
C
G
T
A
C
G
T
A
C
G
T
A
C
G
A
T
C
G
A
T
C
G
A
T
C
G
A
T
C
G
A
T
C
G
T
A
C
T
G
A
C
T
G
A
C
G
T
A
C
G
T
A
C
G
T
A
C
G
T
A
C
G
T
A
G
C
T
A
G
C
T
A
C
G
T
A
C
G
T
A
C
G
T
A
C
T
G
A
C
T
G
A
C
G
T
A
C
G
A
T
C
G
T
A
C
G
T
A
C
G
T
A
C
G
A
T
C
G
T
A
C
G
A
T
C
G
A
T
C
G
A
T
C
G
A
T
C
G
T
A
C
G
T
A
  

|  |  |
| --- | --- |
| p-value: | 1e-22 |
| log p-value: | -5.293e+01 |
| Information Content per bp: | 1.684 |
| Number of Target Sequences with motif | 228.0 |
| Percentage of Target Sequences with motif | 9.87% |
| Number of Background Sequences with motif | 18.0 |
| Percentage of Background Sequences with motif | 5.08% |
| Average Position of motif in Targets | 102.7 +/- 47.4bp |
| Average Position of motif in Background | 117.8 +/- 34.7bp |
| Strand Bias (log2 ratio + to - strand density) | -0.2 |
| Multiplicity (# of sites on avg that occur together) | 1.17 |
| Motif File: | file (matrix) reverse opposite |

### Similar de novo motifs found

|  |  |  |  |  |  |  |  |
| --- | --- | --- | --- | --- | --- | --- | --- |
| Rank | Match Score | Redundant Motif | P-value | log P-value | % of Targets | % of Background | Motif file |
| 1 | 0.658 | G C A T G C T A G C T A G C A T G C A T G C A T C G A T G C A T C G T A G C A T C G A T C G A T G C A T C G A T G C A T G C T A C G T A C G T A C G A T C T G A C G T A G C T A G C T A C G T A C G T A C G T A C G A T C G T A G C T A G C A T G C T A G C T A C G A T C G A T C G A T C G A T C G A T G C A T G C A T C G A T G C A T G C T A C G A T C G A T C G A T | 1e-15 | -36.364330 | 1.60% | 0.52% | motif file (matrix) |
| 2 | 0.664 | G C T A G C T A G C A T G C A T G C A T G C A T G C A T G C T A G C A T G C A T G C A T G C A T C G A T G C A T G C A T G C A T G C A T C G A T C G A T G C A T G C T A G C T A G C A T G C A T G C A T G C A T G C A T G C T A G C A T C G A T G C A T G C A T C G A T C G A T G C A T G C A T G C A T C G A T C G T A G C T A G C T A G C T A G C A T G C T A G C A T G C A T G C T A G C T A G C T A C G T A | 1e-8 | -20.196729 | 1.17% | 0.54% | motif file (matrix) |
